# A randomized controlled study of ureteral stent extraction string on patient’s quality of life and stent-related complications after percutaneous nephrolithotomy in the prone position

**DOI:** 10.1007/s00240-023-01451-5

**Published:** 2023-04-28

**Authors:** Yuanjiong Qi, Hailong Kong, Haonan Xing, Zhihong Zhang, Yue Chen, Shiyong Qi

**Affiliations:** Department of Urology, Tianjin Institute of Urology, The Second Hospital of Tianjin Medical University, Tianjin, 300211 China

**Keywords:** Ureteral stent, Percutaneous nephrolithotomy, Extraction string, Complications, Pain

## Abstract

To demonstrate the Tianjin Institute of Urology (TJIU) technique to place and remove the ureteral stent with extraction string after percutaneous nephrolithotomy (PCNL). Additionally, we aim to compare the pain experienced during stent removal, quality of life during stent retention, and stent-related complications between patients with and without extraction string. 65 patients were included in the final analysis in the string group constructed by the TJIU technique and 66 patients in the conventional double-J ureteral stent (non-string) group. All patients underwent the surgery in a prone position under general anesthesia. They completed the Ureteral Stent Symptom Questionnaire (USSQ) on postoperative days (POD) 7, as well as before their ureteral stent was removed. The visual analogue scale (VAS) pain score (0–10) was completed immediately after the removal of the ureteral stent. Moreover, a specialized person was responsible for recording stent-related complications. All patients completed the USSQ on POD 7, and we did not find a difference in scores in each field. However, there was a significant difference in the “sex” domain before removing the ureteral stent (4.34 vs 3.23; *p* = 0.01). Notably, the use of extraction string after PCNL could decrease the pain associated with stent removal significantly (mean VAS scores 1.45 vs 2.76; *p* < 0.01). Extraction string did not increase the incidence of stent-related complications. We concluded that placing a ureteral stent with an extraction string after PCNL reduces the pain of ureteral stent removal without increasing complications such as accidental removal of the stent, febrile urinary tract infection (UTI).

**Supplementary Information:**

The online version contains supplementary material available at 10.1007/s00240-023-01451-5.

## Introduction

The incidence of urolithiasis is increasing every year, especially in developed countries. Moreover, as one of the high stone prevalence areas, the incidence of nephrolithiasis in China is about 5.8% [1]. In recent years, in addition to traditional open surgery, surgeons can also choose extracorporeal shock wave lithotripsy (ESWL), ureteroscopic lithotripsy (URSL), and percutaneous nephrolithotomy (PCNL) to treat renal calculi with the development of lithotripsy techniques and innovation of equipment [2]. With the higher stone free rates, PCNL is the first-line therapy for large stones, particularly staghorn calculi [3]. During PCNL, it is typically recommended to place a nephrostomy tube and double-J stent to ensure proper urine drainage, facilitate the discharge of residual stones, and assist with compression hemostasis [4]. Recently, with the improvement of PCNL technology and the emphasis of enhanced recovery after surgery, more and more studies have demonstrated that tubeless PCNL (with ureteral stent but without nephrostomy tube) is a safe and ideal surgical approach [5–7]. Nevertheless, the internal double-J stent needs to be removed with a cystoscope in the outpatient clinic, which increases the patient's cost and pain and raises the risk of bleeding, infection, and urethral injury [8]. Nowadays, a ureteral stent is usually equipped with an extraction string for its removal. More than two-thirds of urologists choose to remove the string before indwelling ureteral stents because of concerns about complications associated with extraction string, such as infection, accidental stent dislodgement, and reduced quality of life, especially sexual life [9]. However, previous studies have shown that indwelling a ureteral stent with extraction string after URSL reduces pain during extraction without increasing related complications compared to conventional ureteral stent without extraction string [10–12]. After further reviewing the literature, we found that the removal of the ureteral stent after PCNL by extraction string has not been thoroughly investigated. Therefore, we designed the Tianjin Institute of Urology (TJIU) technique to place and remove the ureteral stent with extraction string after PCNL in the prone position and used a randomized controlled approach to compare the differences in postoperative complications, quality of life, and pain during extubation between string group and non-string group.

## Materials and methods

### Study population

**Fig. 1:**
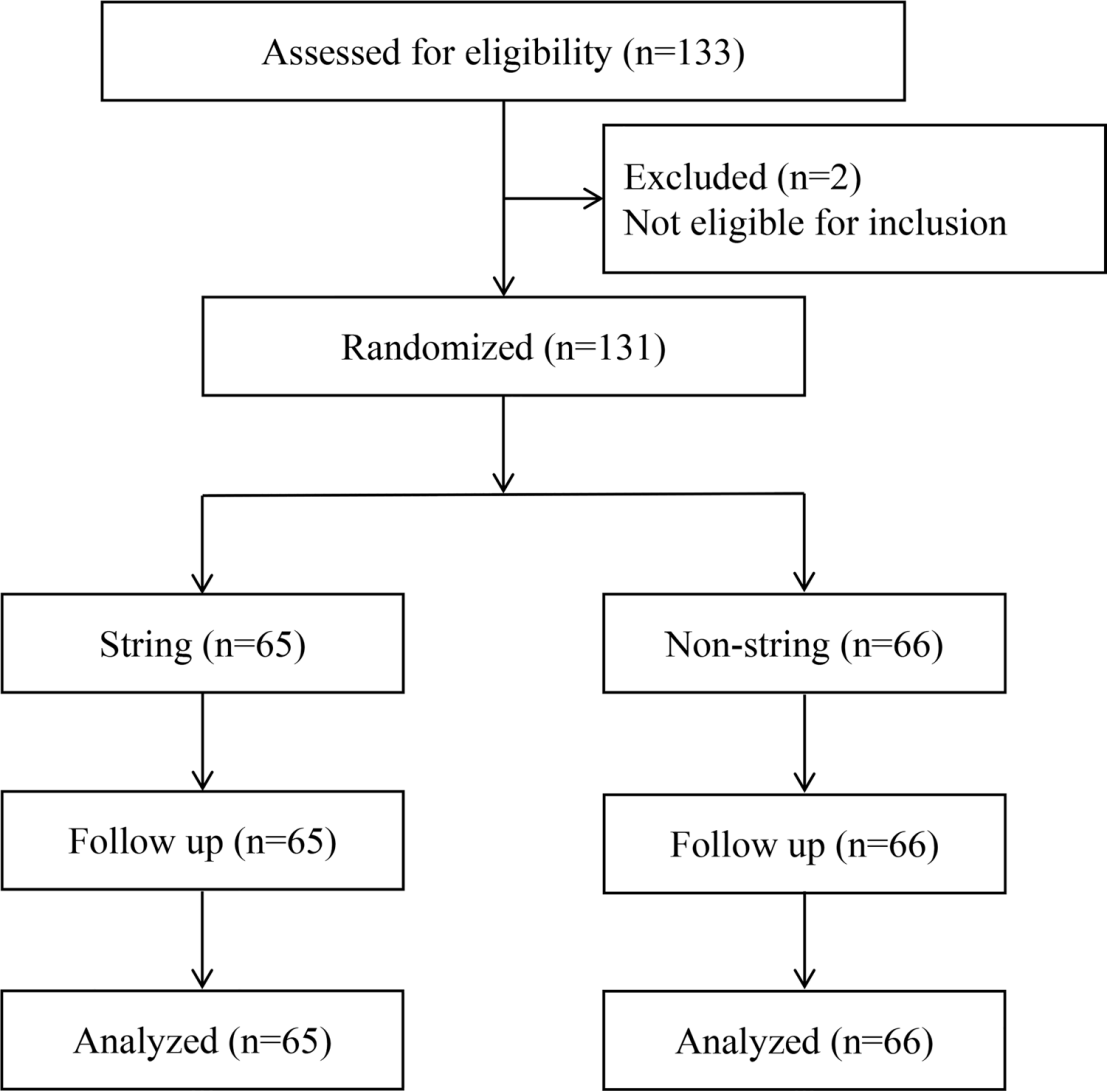
Diagram of study enrollment and final analysis cohort

A total of 133 patients with renal stones who underwent PCNL in our hospital were admitted from September 2021 to September 2022 (Fig. 1). Inclusion criteria: pre-operative diagnosis by urological ultrasound or CT with indications for PCNL. Patients with obstructive factors such as ureteral stenosis preoperatively, pregnancy, inability to obtain informed consent, and presence of psychiatric disorders were excluded. This study was approved by the institutional review board of the Second Hospital of Tianjin Medical University (IRB:21/2022). Patients who met the inclusion criteria were randomized into a string group or non-string group using the “sequentially numbered, opaque, sealed envelopes” method before the surgery.

## Surgical procedures

### Fabrication of ureteral stent and catheter with extraction string

The main primary materials included 6 F double-J stent with extraction string (Soft Percuflex™ Stent with HydroPlus™ Coating; Boston Scientific, MA, USA), 5 F ureteral catheter from Cook Medical (Bloomington, IN, USA), 5 ml syringe, scalpel.

**Fig. 2:**
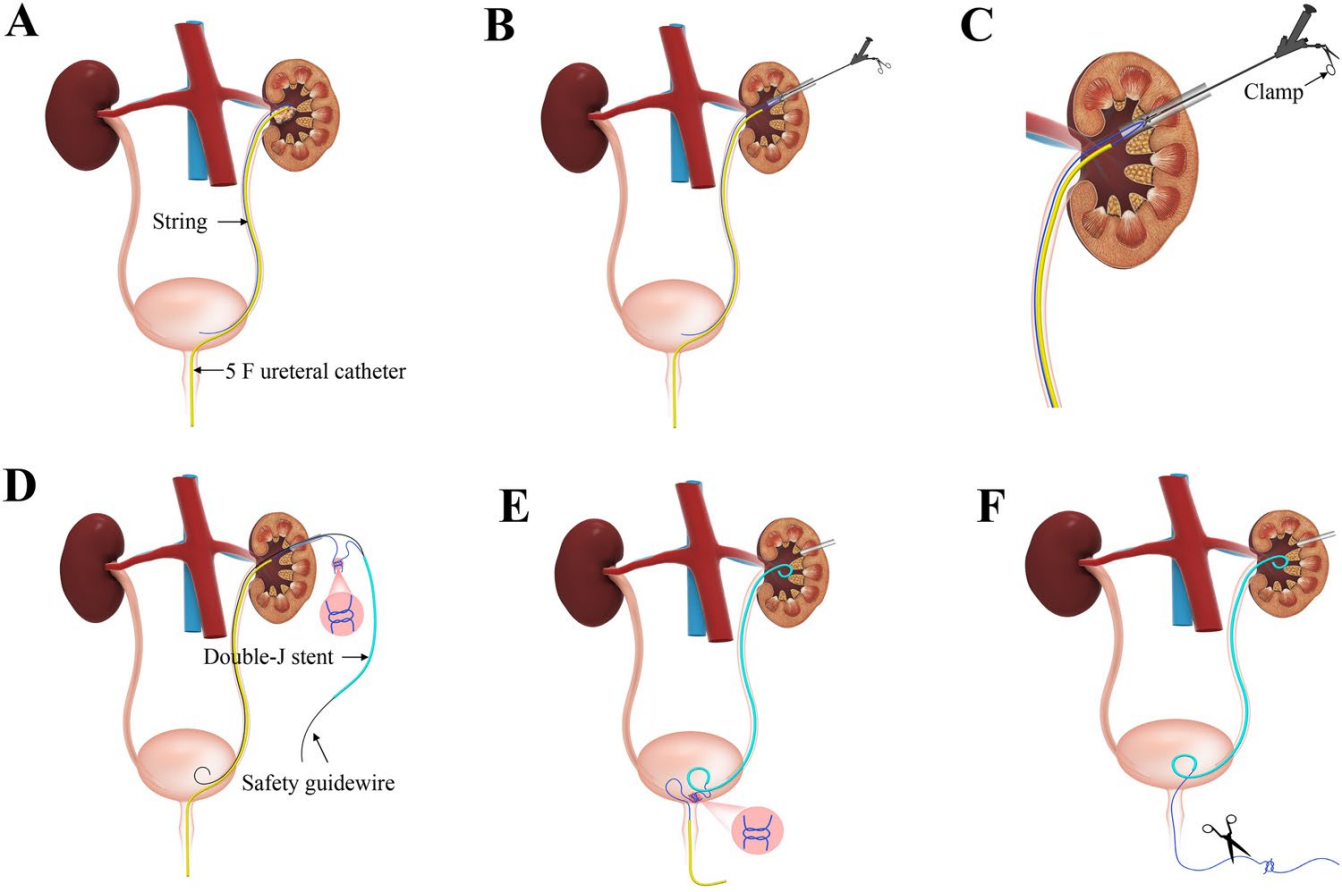
Main steps of TJIU technique: **a** Insert the ureteral catheter with string into ureter. **b** and **c** Take out the string from the renal pelvis with clamp or hook. **d** Tie a knot between the strings of ureteral catheter and double-J stent. **e** Remove ureteral catheter while placing the double-J stent anterogradely under the guidance of safety guidewire. **f** Cut off the string approximately 6–8 cm from the external urethral orifice

The extraction string at the end of the double-J stent was snipped from the knot in the string group. Part of the string was fixed at the distal end of the double-J stent (35 cm for the male patient and 25 cm for the female patient) (Fig. 1), and the residual part was secured at the head of the 5 F ureteral catheter (approximately 30 cm in length) with a 5 ml syringe needle (Fig. 2).

### Percutaneous nephrolithotomy

Firstly, the patient was placed in the lithotomy position after general anaesthesia. The ureteral catheter was then inserted in the renal pelvis under the surveillance of the 8/9.8 F ureteroscope along a safety guidewire, attached to a sterile urethral catheter and linked to saline to create artificial hydronephrosis (Fig. 2a).

Secondly, the patient was placed in a prone position. Under the guidance of a 3.5-MHz ultrasound probe (MEDISON Ultrasound System), a mini (18F) or standard (24F) percutaneous tract was established. Then, under the supervision of an 8/9.8 F ureteroscope or 20.8F nephoscope, stones were fragmented and cleared using a combination of ultrasonic and pneumatic lithotripter (LithoClast Master; EMS Electro Medical Systems) or holmium:YAG laser (SRM-H2B, raykeen, Shanghai, China). In the string group, it was essential to protect the string from accidental breaking if it appeared in the surgeon's visual field.

**Fig. 3:**
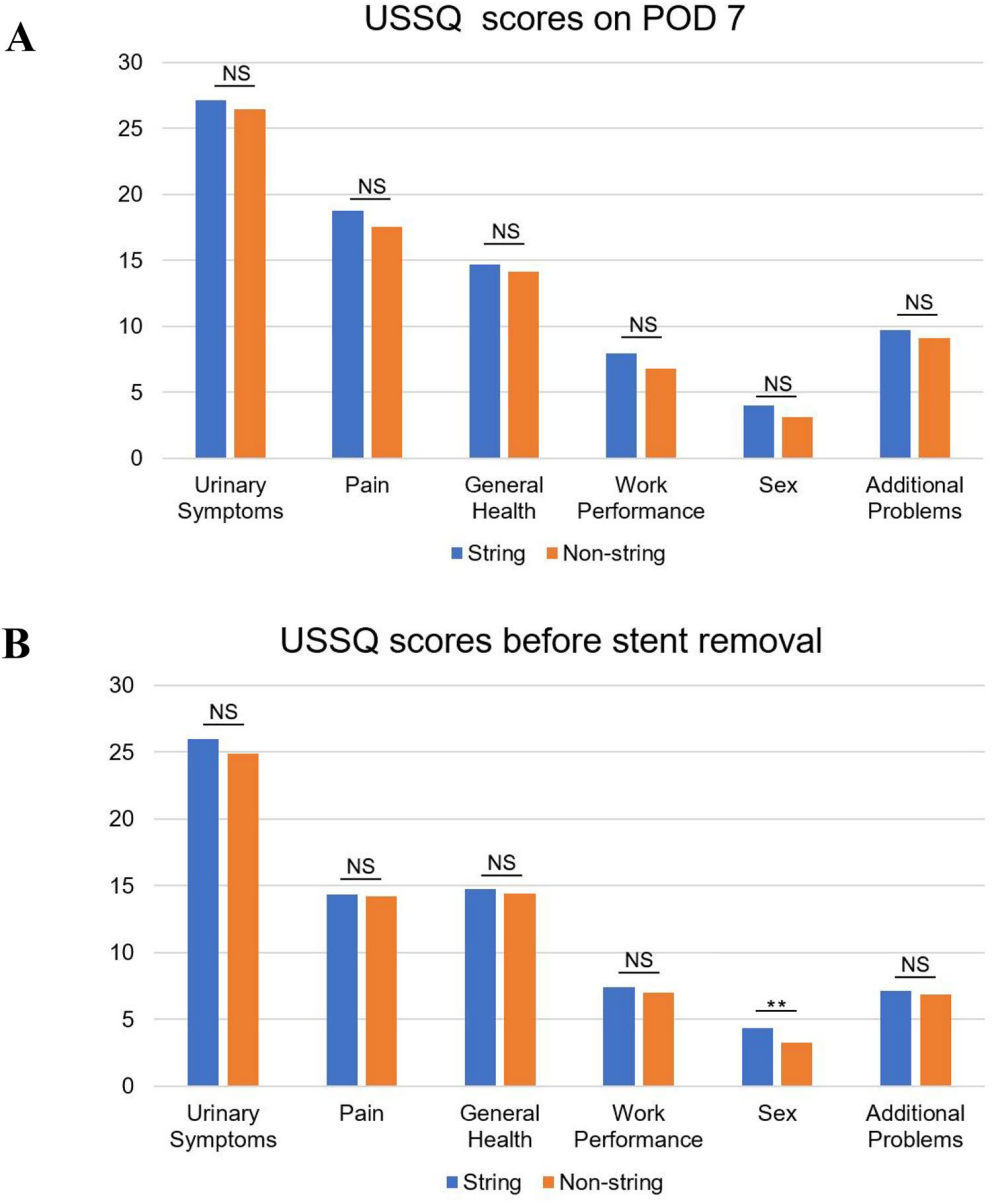
Ureteral Stent Symptom Questionnaire (USSQ) domain scores: **a** Postoperative day 7 (POD 7). **b** Before stent removal. NS, no significance; **p-value ≤ 0.01

Thirdly, once the stone was fragmented and cleared, we took out the string (Figs. 3) fixed to the head of the 5 F ureteral catheter with a clamp or hook (Fig. 2b, c) in the string group and tied it to the string on the double-J stent (Fig. 2d). Subsequently, we inserted the safety guidewire through the double-J stent into the ureter anterogradely. Then, the assistant slowly pulled out the ureteral catheter completely from the urethral orifice while the surgeon placed the double-J stent anterogradely under the guidance of safety guidewire. In the non-string group, we removed the extraction string and used the antegrade method to place the double-J stent, which was inserted along a safety guidewire in the ureter. After inserting the stent, we confirmed the position of the double-J stent in the renal pelvis and checked for any bleeding in the percutaneous renal tract. The nephrostomy tube was subsequently inserted in all patients.

Finally, the patient was placed in a supine position. In the string group, the string was gradually withdrawn outward from the external urethral orifice, and the knot was snipped once it was visible, approximately 6–8 cm from the external urethral orifice (Fig. 2e, f). The string group patients were all informed of the extraction string's purpose and attention points during hospitalization.

### Removal of the ureteral stent

The ureteral stent was removed from all patients within 2–4 weeks postoperatively. For the string group, we removed the ureteral stent by pulling the extraction string out directly. The ureteral stent in non-string group was removed using rigid cystoscopic procedures in lithotomy position, with oxybuprocaine hydrochloride gel being applied to the urethra.

### Postoperative follow-up

In both groups, the Ureteral Stent Symptom Questionnaire (USSQ) was completed on postoperative days (POD) 7 and the day when stent was removed. It is made up of several domains such as “urinary symptoms”, “pain”, “general health”, “work performance”, “sex”, and “additional problems”. The visual analogue scale (VAS) pain score (0–10) was completed immediately after the removal of the ureteral stent. In addition, a designated individual was responsible for recording stent-related complications such as febrile UTI (> 38℃), emergency room (ER) visits, accidental stent dislodgement, and delayed removal. Urological ultrasound or CT was used to assess the presence of residual stones (> 4 mm) one month after surgery.

### Statistical analysis

The primary endpoint of our study was the VAS pain scores at stent removal. The sample size was calculated using the PASS software based on the results of previous ureteral stent study with a power of 90% and a type-1 error (α) of 0.05. The number of participants was increased to account for patient loss to follow-up and withdrawals [12]. R × 64 4.1.2 statistical software was used to process the data. The *t*-test was used for comparison between groups of continuously normally distributed measurement data, and the rank sum test was used for counting data that did not conform to the normal distribution. The χ^2^ test was performed for comparison between groups of counting data. The difference was considered statistically significant at *P* values < 0.05.

## Results

A total of 131 patients met the inclusion criteria. They were randomly assigned to either the string (*n* = 65) or the non-string group (*n* = 66) (Fig. 1). There was no significant difference in gender ratio, age, BMI (Body Mass Index), stone size, and stone-free rate (*p* > 0.05). However, the postoperative stenting periods were longer in the non-string group (*p* < 0.01; Table 1).

**Table 1:** Baseline characteristics of patient

Variables	String	Non-string	*P*^a^
Total number of patients, *n*	65	66	
Gender, *n* (%)			
Male	49 (75.38)	40 (60.61)	0.10
Female	16 (24.62)	26 (39.39)	
Mean age ± SD, years	56.38±9.69	54.71±12.50	0.39
Mean BMI ± SD, kg/m^2^	26.10±3.36	25.66±3.70	0.47
Stone side, *n* (%)			
Left	43 (66.15)	39 (59.09)	0.51
Right	22 (33.85)	27 (40.91)	
Stone location, *n* (%)			
Renal	48 (73.85)	48 (72.73)	0.65
Ureteric	5 (7.69)	8 (12.12)	
Both	12 (18.46)	10 (15.15)	
Mean stone size ± SD, mm	38.15±16.44	33.23±14.49	0.07
Mean postoperative stenting periods ± SD, weeks	2.00±0.31	2.27±0.65	0.003
Stone-free rate, *n* (%)	78.46	72.31	0.45

^a^Pearson’s chi-squared, Student *t* test

All patients completed the USSQ on POD 7, and we did not find a statistical difference in scores in each field (*p* > 0.05; Fig. 3a, details in Table S1). Before removing the ureteral stent, all patients completed the questionnaire again. Surprisingly, there was no difference between the two groups in “urinary symptoms”, “pain”, “general health”, “work performance”, and “additional problems”. However, there was a statistically significant difference in “sex”, as shown in Fig. 3b (4.34 vs. 3.23; *p* = 0.01; details in Table S2).

Table 2 showed the VAS pain scores of the patients during the removal of the ureteral stent. Overall, the mean pain score was 1.45 in the string group and 2.76 in the non-string group, indicating a statistically significant difference (*p* < 0.01). When compared within groups, male patients scored significantly higher than females in the non-string group (*p* < 0.05).

**Table 2:** VAS pain scores on ureteral stent removal

VAS score	String (*n*=65)		Non-string (*n*=66)		*P*^*b*^
	Mean (SD)		Mean (SD)		
Overall	1.45	(0.64)	2.76	(1.01)	<0.01
Male	1.53	(0.62)	2.98	(1.03)	<0.01
Female	1.19	(0.66)	2.42	(0.90)	<0.01
*P*^a^	0.08		0.03		

^a^Male and female in the same group

^b^Between groups with and without string

We recorded the complications associated with stent in both groups (Table 3). In the string group, one female patient (1.5%) accidentally removed the ureteral stent after urination. Another female patient developed a febrile UTI (38.1℃) and improved after taking antibiotics. Two male patients (3%) called the doctor to consult whether they could take oral medication for pain relief. In the non-string group, one patient presented to the emergency department with a febrile UTI (38.2℃). The retention time of the stent had to be extended for two patients in the non-string group due to the lack of sufficient sterilized cystoscopes. However, the ureteral stent in the string group was removed either by a physician in the hospital outpatient clinic or by themselves under the guidance of a physician. In summary, there was no statistically significant difference in stent-related complications between the two groups (*p* > 0.05).

**Table 3:** Complications associated with stent

Complications (*n*, %)	String (*n*=65)	Non-string (*n*=66)	*P*^a^
Overall	4	3	0.72
Male	2	2	
Female	2	1	
Febrile UTI	1	1	1.00
Emergency room visits	0	1	1.00
Accidental removal	1	0	0.50
Stent dislodgement	0	0	–
Phone calls	2	0	0.24
Delayed removal	0	2	0.50

^a^Fisher’ s exact test

## Discussion

Urolithiasis is a common urological condition. With the development of technology, PCNL has become the preferred treatment for kidney and upper ureteral stones larger than 2 cm, particularly staghorn calculi [13, 14]. It has the advantages of high treatment efficiency and minimal invasion [15]. Traditionally, the nephrostomy tube and ureteral stent are placed after PCNL. The nephrostomy tube has several benefits: (I) draining urine adequately and preventing the occurrence of urinary extravasation; (II) providing pressure on the nephrostomy tract for hemostasis meanwhile observing whether there is active bleeding; (III) performing staged surgery from the tract for residual stones [13, 16, 17]. Moreover, the double-J stent is equally essential considering its function: (I) prevention of postoperative ureteral oedema and stricture; (II) promotion of the residual stones removal and adequate internal drainage [18–20]. Recently, tubeless PCNL has been investigated, mainly including standard tubeless (postoperative placement of ureteral stent only) and totally tubeless (neither ureteral stent nor nephrostomy tube) [5, 21]. Numerous studies have demonstrated that the tubeless PCNL (ureteral stent only) technique is being employed with increasing frequency. It has been reported to be both safe and effective, as well as associated with a shorter hospital stay and a reduced incidence of postoperative pain [6, 22, 23]. Nevertheless, completely tubeless PCNL technique (neither ureteral stent nor nephrostomy tube) is still challenging and must also be performed by experienced surgeons [7, 24–26].

Regardless of traditional PCNL (placing both nephrostomy and ureteral stent) or tubeless PCNL (ureteral stent only), the ureteral stent needs to be removed by rigid or flexible cystoscope in the outpatient setting after surgery. To reduce the morbidity caused by cystoscopic extubation, Agrawal et al. and Shpall et al. tied a string to the proximal end of the double-J stent, and the string was fixed to the skin surface through the nephrostomy tract [19, 27]. Nevertheless, this method could lead to unforeseen consequences such as: (I) a large amount of urine leaked from the tract after surgery, which increased the risk of urinary extravasation and frequent changes of wound dressings; (II) the extraction of the double-J stent from the nephrostomy tract increases the risk of infection and bleeding.

Preserving the extraction string equipped on the ureteral stent could solve those problems. Some urologists are concerned that ureteral stent with extraction string may affect patients' quality of life, increase the incidence of infection, lead to premature migration or dislodgement of the stent. Nevertheless, ureteric stent placement with extraction string has been well-studied in URSL. In a prospective randomized controlled study, Barnes et al. demonstrated that ureteral stent with extraction string offered many advantages and did not increase stent-related urologic symptoms, complications, or postoperative morbidity [11]. Freifeld et al. also found no statistically significant difference in overall infection rates between the group with and without string by a retrospective study [28]. Concerning the cost–benefit between with and without extraction string, Liu et al. found that stent with extraction string provided significant cost savings for patients [29]. Removal of the ureteral stent with extraction string was significantly less painful than with cystoscope (mean VAS scores 5.67 vs 2.73; *p* < 0.001) [12]. In a recent study, Shah et al. compared stent-related complications with the placement of a conventional ureteral stent and a complete intra-ureteric stent with extraction string and showed that no difference in complication rates between the two groups [30].

Similar studies have rarely been performed in PCNL. To assess the safety and efficacy of placing the ureteral stent with extraction string after PCNL in the prone position, we designed this study using the TJIU technique and obtained similar outcomes. The USSQ scores for each domain on POD 7 between the two groups of patients showed no significant difference in the results of our study. Before the ureteral stent removal, the scores were higher in the non-string group than the string group in most domains, although the difference between them was not statistically significant. Except that the score of the “sex” domain was significantly higher in the string group (4.34 vs 3.23; *p* = 0.01). Because some patients were concerned about accidental premature removal of the ureteral stent during sexual intercourse, this concern seems to be more common in male patients. Consistent with the results of previous studies, our data analysis showed that VAS scores were significantly higher in the non-string group (mean VAS scores 2.76 vs 1.45; *p* < 0.01). This indicates that ureteral stent removal by extraction string can significantly reduce patients' pain during the process. Furthermore, the extraction string did not increase the overall incidence of febrile UTI during indwelling ureteral stent [12, 28]. The same results were found in our study (*p* = 1.00). Shah et al. reported a 5% rate of stent migration and accidental dislodgement [30]. In the present study, we found that 1.5% (one female) of patients with an extraction string prematurely removed the ureteral stent. This patient accidentally removed the stent while wiping her urethra with toilet paper after urination. There are many indications for cystoscopy, including either visible or microscopic blood in the urine, as a surveillance method after bladder cancer, removal of the ureteral stent, etc. As a result, two patients had to prolong ureteral stent retention because the outpatient cystoscopy was fully occupied with appointments. Patients with extraction string can also remove the ureteral stent themselves at home under medical supervision. It reduces the cost of transportation and cystoscopic extraction (approximately $62.86 in our center). As shown by Liu et al., patients with extraction string cost significantly less than patients who were extubated via cystoscope ($12.82 vs $75.12; *p* = 0.008) [29]. It also reduces the risk of exposure and infection during the Corona Virus Disease 2019 (COVID-19) outbreak. In summary, ureteral stent removal by extraction string after PCNL could reduce healthcare costs, decrease pain associated with stent removal, and improve patient convenience without increasing stent-related complications.

There are some limitations in our study. Firstly, the sample size was relatively small and it was a single-center prospective study. Secondly, we did not statistically analyze the cost differences between the two groups of patients in detail. Finally, the TJIU technique was only applied to PCNL in the prone position in the present trial. Because we could insert the ureteral stent with extraction string retrogradely into the ureter in several selected patients who underwent PCNL combined with retrograde flexible ureteroscopy in the supine lithotomy position.

## Conclusion

Placing a ureteral stent with extraction string following TJIU technique after PCNL in prone position could reduce the pain associated with ureteral stent removal, and avoid complications such as urethral injury caused by cystoscopic ureteral stent removal, while not increasing complications related to accidental removal and febrile UTI. Therefore, a ureteral stent with extraction string is feasible for PCNL patients, whereas it should be chosen cautiously for sexually active patients.

## Supplementary Information

Below is the link to the electronic supplementary material.

Supplementary file1 (TIF 475 KB)

Supplementary file2 (TIF 366 KB)

Supplementary file3 (TIF 331 KB)

Supplementary file4 (PPT 141 KB)

Supplementary file5 (MP4 144821 KB)

## Data Availability

The datasets generated and analyzed during the current study are available from the corresponding author on reasonable request.
