# Supplementary figures and images for: A randomized controlled study of ureteral stent extraction string on patient’s quality of life and stent-related complications after percutaneous nephrolithotomy in the prone position
Source: Urolithiasis. 2023 Apr 28;51(1):79. doi: 10.1007/s00240-023-01451-5 (PMC10141830; doi:10.1007/s00240-023-01451-5)

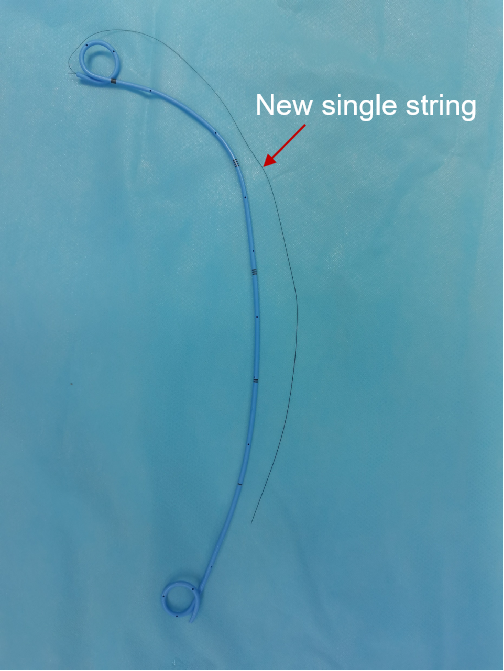

Supplement: Supplementary file 1 — Supplementary file1 (TIF 475 KB) [file 240_2023_1451_MOESM1_ESM.tif]

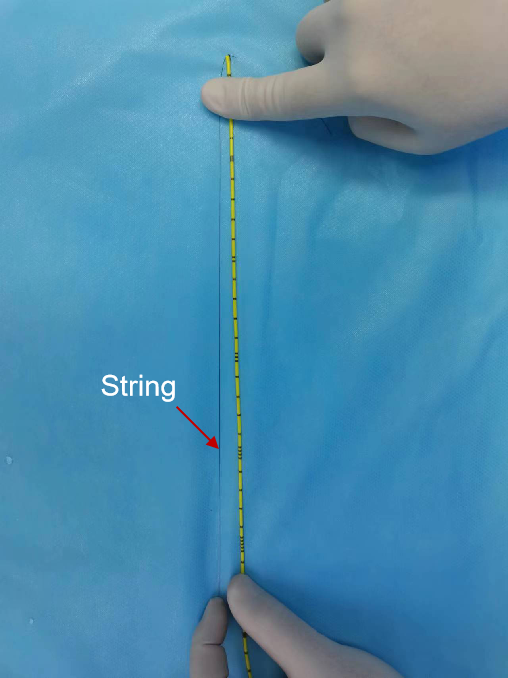

Supplement: Supplementary file 2 — Supplementary file2 (TIF 366 KB) [file 240_2023_1451_MOESM2_ESM.tif]

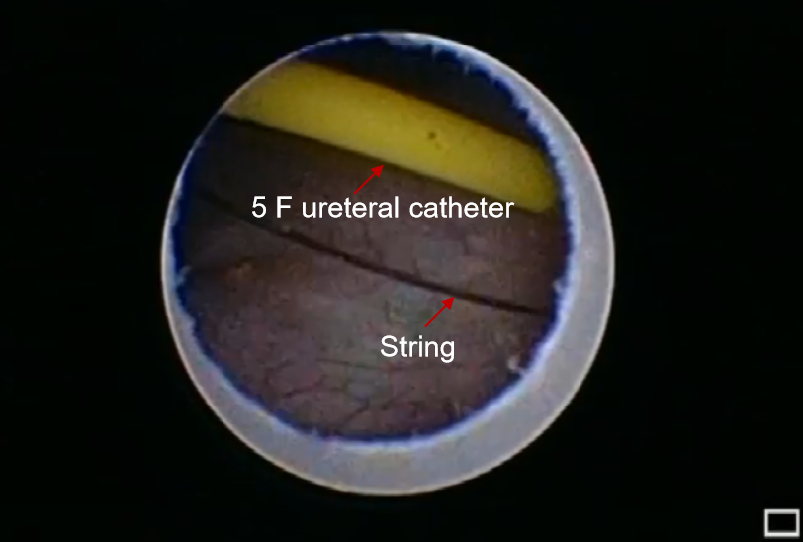

Supplement: Supplementary file 3 — Supplementary file3 (TIF 331 KB) [file 240_2023_1451_MOESM3_ESM.tif]
