# Supplementary material for: A randomized controlled study of ureteral stent extraction string on patient’s quality of life and stent-related complications after percutaneous nephrolithotomy in the prone position
Source: Urolithiasis. 2023 Apr 28;51(1):79. doi: 10.1007/s00240-023-01451-5 (PMC10141830; doi:10.1007/s00240-023-01451-5)
Supplement: Supplementary file 4 — Supplementary file4 (PPT 141 KB) [file 240_2023_1451_MOESM4_ESM.ppt]

## Slide 1
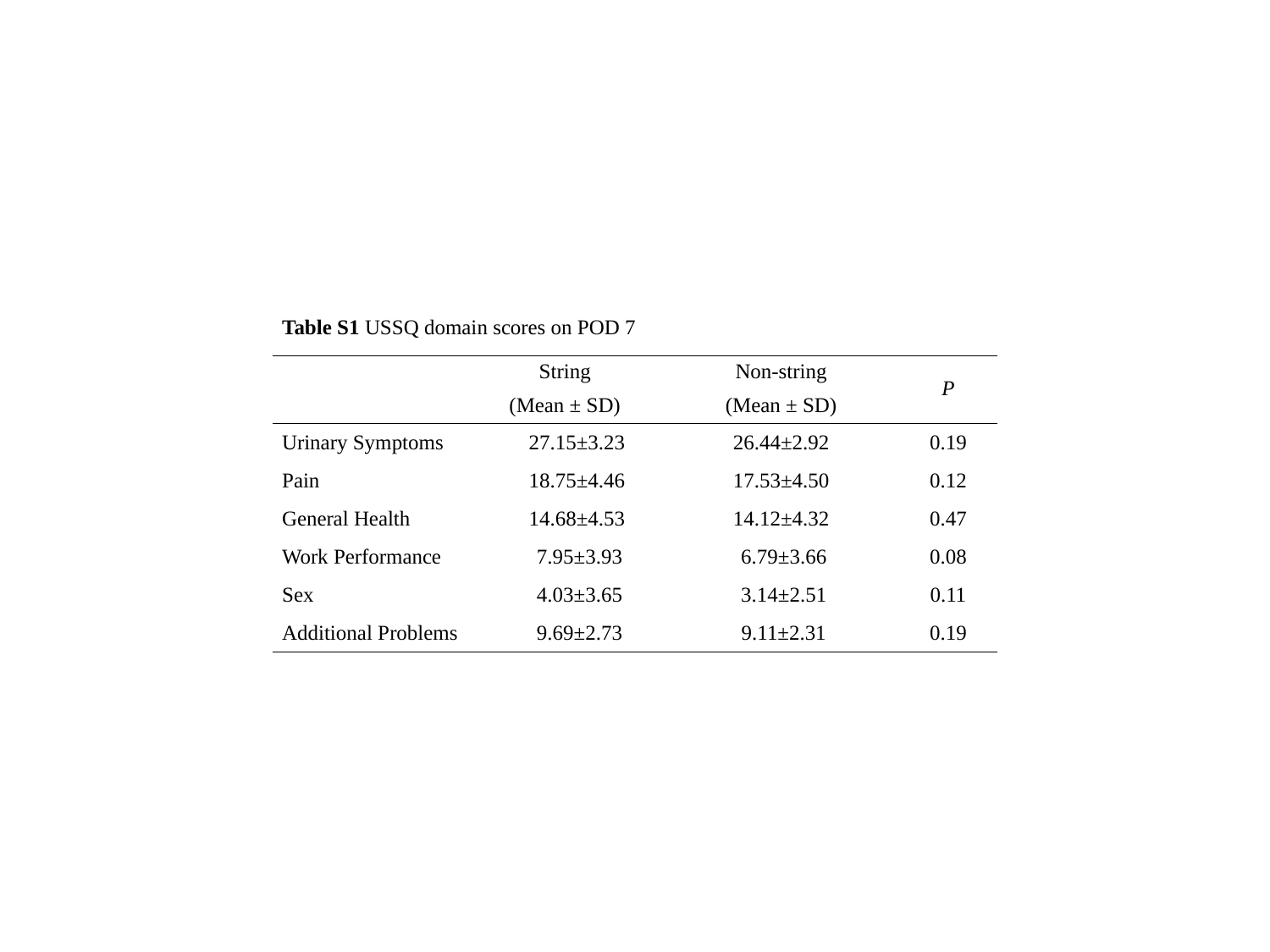

| Table S1 USSQ domain scores on POD 7 | | | | |
| --- | --- | --- | --- | --- |
| | String | | Non-string | P |
| | (Mean ± SD) | | (Mean ± SD) | |
| Urinary Symptoms | | 27.15±3.23 | 26.44±2.92 | 0.19 |
| Pain | | 18.75±4.46 | 17.53±4.50 | 0.12 |
| General Health | | 14.68±4.53 | 14.12±4.32 | 0.47 |
| Work Performance | | 7.95±3.93 | 6.79±3.66 | 0.08 |
| Sex | | 4.03±3.65 | 3.14±2.51 | 0.11 |
| Additional Problems | | 9.69±2.73 | 9.11±2.31 | 0.19 |

## Slide 2
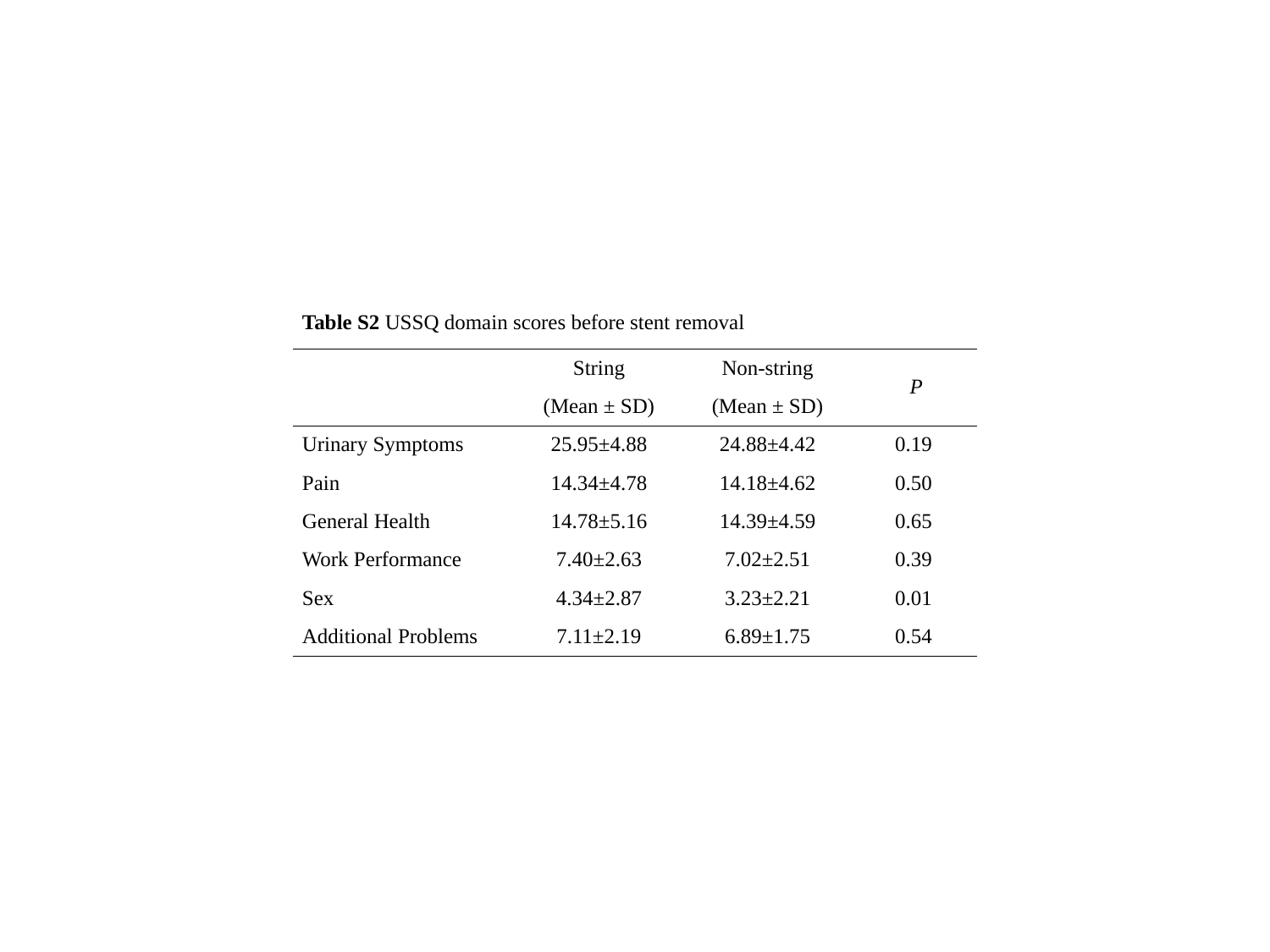

| Table S2 USSQ domain scores before stent removal | | | |
| --- | --- | --- | --- |
| | String | Non-string | P |
| | (Mean ± SD) | (Mean ± SD) | |
| Urinary Symptoms | 25.95±4.88 | 24.88±4.42 | 0.19 |
| Pain | 14.34±4.78 | 14.18±4.62 | 0.50 |
| General Health | 14.78±5.16 | 14.39±4.59 | 0.65 |
| Work Performance | 7.40±2.63 | 7.02±2.51 | 0.39 |
| Sex | 4.34±2.87 | 3.23±2.21 | 0.01 |
| Additional Problems | 7.11±2.19 | 6.89±1.75 | 0.54 |
